# Immune-related mechanisms of fecal microbiota transplantation in the intestinal microenvironment as a potential intervention for autism spectrum disorder patients

**DOI:** 10.3389/fphar.2026.1775104

**Published:** 2026-07-08

**Authors:** Orleancio Gomes Ripardo de Azevedo, Paulo Roberto Leitão de Vasconcelos, Paulo Nazareno Soares Rosa, Gabriella Cunha Vieira Ciurleo, Cirle Alcantara Warren, Reinaldo Barreto Oriá

**Affiliations:** 1 Department of Morphology and Institute of Biomedicine, Laboratory of the Biology of Tissue Healing, Ontogeny and Nutrition, School of Medicine, Federal University of Ceara, Fortaleza, Brazil; 2 Department of Surgery, School of Medicine, Federal University of Ceara, Fortaleza, Brazil; 3 Division of Infectious Disease and International Health, University of Virginia, Charlottesville, VA, United States; 4 Department of Morphology, Faculty of Medicine, Federal University of Ceara, Fortaleza, Brazil

**Keywords:** autism spectrum disorder, dysbiosis, fecal microbiota transplantation, gut–brain axis, neuroinflammation

## Abstract

Autism spectrum disorder (ASD) is a complex neurodevelopmental condition characterized by behavioral, cognitive, and motor impairments. There is increasing evidence linking ASD with an altered composition of the gut microbiota and chronic low-grade inflammation, suggesting a key role of the gut–brain axis (GBA) in the pathophysiological development of this condition. This mini review explores the molecular and immunological mechanisms underlying the associations between ASD and gut dysbiosis, with particular emphasis on the therapeutic potential of fecal microbiota transplantation (FMT). Dysbiosis can compromise the integrity of the intestinal barrier, increasing permeability and the translocation of pathogen-associated molecular patterns (PAMPs), such as lipopolysaccharides (LPS), thereby releasing inflammatory cytokines, including IL-6 and TNF-α. These mediators activate the mucosal immune pathways, such as the NF-κB signaling and NLRP3 inflammasome, thereby contributing to neuroinflammation and elevating intestinal biomarker levels, such as S100B, RANTES, and calprotectin. Emerging evidence suggests that FMT may restore microbial diversity, promote the expansion of beneficial short-chain-fatty-acid-producing taxa, and reinforce intestinal tight junction proteins, thereby improving the integrity of the gut barrier. These effects may attenuate systemic inflammation, modulate central immune responses, regulate neurotransmitter levels, and improve gastrointestinal and behavioral outcomes in individuals with ASD. Despite these promising findings, current evidence remains limited by small sample sizes, methodological heterogeneity, and short follow-up periods. Hence, future research efforts should prioritize well-designed randomized controlled trials and the development of personalized microbial-based interventions to establish FMT as a safe and effective therapeutic strategy for ASD.

## 1 Background

The gastrointestinal (GI) tract is one of the largest interfaces between an organism and its environment. There are numerous microorganisms associated with the risk of disruption of the intestinal mucosal barrier integrity (Aburto and Cryan, 2024).

The microorganisms colonizing the gut mucosa are collectively called the “gut microbiota,” which constitutes a rich and diverse ecosystem with several intricate molecular and beneficial interactions with itself and the host (Khan et al., 2025; Gillingham et al., 2025).

A previous study estimated that microbiota comprise more cells than the mammalian host; however, studies have suggested that the ratio of these eukaryotic cells is approximately 1:1 (Sender et al., 2016). However, the genome from the gut-based ecosystems, i.e., bacteria, fungi, and viruses that collectively form a microbiome, vastly exceeds that of the host genome (Moeller, 2021).

The interactions between the microbiota and gut immune cells are associated with various benefits to the host, including gut barrier integrity, improved intestinal mucosal architecture (Kayama et al., 2020), energy metabolism (Bohan et al., 2019), and host immune regulation against pathogens (Bäumler and Sperandio, 2016; Gensollen et al., 2016).

These balanced mechanisms can be disrupted by modifications to the diversity of microbiota, which is known as “dysbiosis.” The terms “eubiosis” and “dysbiosis” were initially introduced at the beginning of the 20th century in the context of microbial ecology and host–microorganism interactions. A historical review by Hooks and O’Malley highlighted the seminal contributions of the microecologist Helmut Haenel, whose work helped to shape the modern conceptualization of microbial equilibrium and imbalance in human health. The review further argues that dysbiosis encompasses not only taxonomic alterations in microbial communities but also functional disruptions in host–microbiota interactions associated with disease processes (Hooks and O’Malley, 2017).

The gut mucosal environment establishes a complex network of molecular interactions between bacterial cells and the mucosal epithelium to regulate the immune system, thereby helping control over pathogenic strains (Yoo et al., 2020; Wang et al., 2024; Iliev et al., 2025). Additionally, there is growing evidence associating the pro-inflammatory patterns of dysbiosis in the intestinal environment with several disorders (Chang and Lin, 2016; Schroeder and Bäckhed, 2016).

**FIGURE 1:**
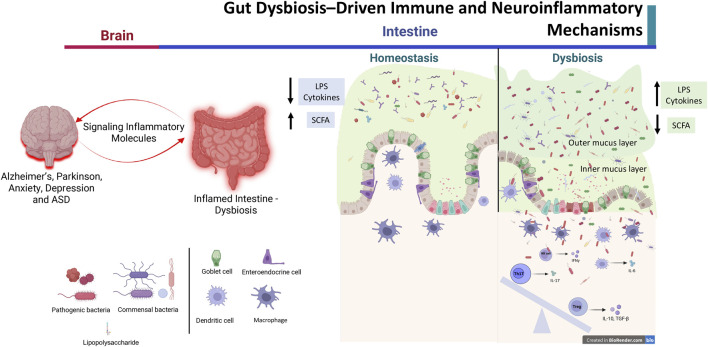
This schematic illustrates the mechanisms linking gut microbiota dysbiosis to systemic inflammation and central nervous system (CNS) dysfunction. Under physiological conditions, the gut microbiota maintains mucosal homeostasis, epithelial integrity, and immune regulation. Dysbiosis promotes immune imbalance, characterized by altered Th1/Th17 and Treg responses, increased production of pro-inflammatory cytokines, such as IL-6 and IFN-γ, and enhanced activation of innate immune pathways. These inflammatory processes contribute to blood–brain barrier signaling, leading to microglial activation and neuroinflammation. Consequently, alterations in neurotransmitter systems may occur, ultimately affecting neuronal function and behavior. Collectively, these pathways highlight the role of gut dysbiosis in the pathophysiology of neurodevelopmental disorders, including autism spectrum disorder (ASD), through the gut–brain axis. Created in Biorender.com.

The intestinal microbiota can affect the central nervous system (CNS) by synthesizing and releasing neurotransmitters, amino acids, and other microbial metabolites. When the gut barrier is compromised, the microbial-derived molecules can translocate across the epithelial barrier to interact with the host immune cells and metabolic systems. This disruption affects the regulatory function of the barrier in controlling the passage of luminal substances into circulation, ultimately influencing the homeostasis of the CNS (Oriá et al., 2016; McCormick et al., 2020). The translocation of LPS can trigger systemic immune activation and neuroinflammatory cascades (Huo et al., 2021) (Figure 1).

Alzheimer’s disease, Parkinson’s disease, anxiety, depression, and, more recently, autism spectrum disorder (ASD) are complex conditions of the CNS that are associated with a pro-inflammatory background (Wang et al., 2018; Tylutka et al., 2024).

ASD is defined as a complex and multifactorial neurodevelopmental disorder that negatively affects individuals in social interactions, including communication skills and cognitive, motor, and behavioral patterns; it also exhibits significant differences among individuals (Qin et al., 2024). There are several genetic and environmental aspects associated with the onset of ASD and its outcome levels. However, there is no single study on ASD detailing a clear cause-and-effect association or even identifying trustworthy and consistent biomarkers. Recently, several aspects have been discussed as factors leading to the increasing prevalence of ASD globally. The pro-inflammatory framework is known to contribute to the onset of ASD, including activation of the microglia, higher levels of pro-inflammatory chemokines, and modification of immune responses (Prata et al., 2019).

Additionally, advances in research on molecular interactions between the microbiome and the host have highlighted the gut mucosa as a key interface where microbial signals are translated into systemic immune responses, thereby influencing the maintenance or disruption of mucosal homeostasis and overall health of the gut–brain axis (GBA) (Zhuang et al., 2024; Zhou et al., 2025).

In the present narrative review, we aim to discuss the associations between dysbiosis and ASD; we further examine how fecal microbiota transplantation (FMT) could be an interesting option in addressing the pro-inflammatory framework of molecular–immune mechanisms involved in the disrupted gut mucosal barrier and its interactions with the commensal and pathogenic microorganisms.

## 2 Dysbiosis, inflammation, and ASD

Dysbiosis is described as the activation and recruitment of inflammatory cells, resulting from an imbalance of the commensal and pathogenic communities of microorganisms in the intestinal tract (Liu et al., 2019).

This imbalance of the microorganism ecosystem reduces the microbial diversity by lowering the beneficial taxa and increasing the pathogenic populations, resulting in the induction of tissue–mucosal modifications that lead to intestinal barrier damage, increased intestinal inflammation, and permeability to several molecules (“leaky gut”), which are associated with several systemic inflammation and CNS disorders (Pellegrini et al., 2023).

Recent studies have linked dysbiosis with a variety of disorders of the CNS, including Alzheimer’s disease (Borsom et al., 2020; Abdol Samat et al., 2025; Kozhakhmetov et al., 2024), Parkinson’s disease (Berthouzoz et al., 2023; Munoz-Pinto et al., 2024; Barichella et al., 2019), anxiety (Nikel et al., 2025), depression (Lin et al., 2025), and ASD (Alharthi et al., 2022; Zarimeidani et al., 2024; Yadav et al., 2025).

Dysbiosis is associated with the release of several key immune-related pro-inflammatory mediators, such as IL-6, IL-1β, TNF-α, and IFN-γ (Nusrat et al., 2000; Ma et al., 2004). This alters the production of microbial metabolites, such as SCFAs, and pathogen-associated molecular patterns (PAMPs), such as LPS, which can cross the compromised intestinal barrier and affect brain function via the bloodstream or the vagus nerve, thereby initially influencing the risk of ASD (Saresella et al., 2016).

It has been found that the microbiomes of children with ASD differ significantly from those of healthy individuals (West et al., 2022). Dysregulation of the intestinal barrier, brain’s neurotransmitter levels, and microglia activation have been highlighted as important drivers of neuroinflammation in ASD (Doenyas, 2018; Siniscalco et al., 2018). It is suggested that the imbalance of pathogenic microbiota could lead to a pro-inflammatory framework and affect how the gut microbiota drive the GBA in individuals with ASD.

Studies on gut dysbiosis and inflammation have revealed that dysbiotic individuals with ASD are significantly prone to activation of the inflammatory and immune pathways, including the release of IL-2, 6, and 12 and the activation of the toll-like receptor (TLR) 3 (Hughes et al., 2023; Guevara-Ramírez et al., 2025). Moreover, increased activation of the NLRP3 inflammasome, elevated levels of IFN-γ, and enhanced NF-κB signaling may serve as markers of dysbiotic-driven mucosal inflammation, accompanied by altered Th-17/T-Reg cell ratios and imbalances in macrophage activation mediated by TNF-α, IL-1β, IL-18, and IL-6 (Carloni and Rescigno, 2023; Zhao et al., 2023). A general overview of the intestinal dysbiosis-driven immune-inflammatory mechanism is shown in Supplementary Figure S1.

The association between immune dysregulation and ASD onset/severity has also been discussed in the literature (Than et al., 2023; Usui et al., 2023), which documented increased plasma levels of pro-inflammatory molecules (TNF-α, IFN-γ, and IL-2, IL-4, IL-5, IL-6, IL-8, IL-17, and IL-10) in individuals with ASD (Naranjo-Galvis et al., 2026; Cao et al., 2021; Ye et al., 2021).

Several studies have investigated the biomarkers of immune inflammatory activation involving the intestinal mucosa and CNS in individuals with ASD and their impacts on the induction of immune-modulating cascades. The S100B subunit of a calcium-binding protein is expressed in astrocytes and enteric glial cells; there is evidence demonstrating a positive association between peripheral levels of S100B and neuroinflammation in individuals with ASD, suggesting a potential role in ASD onset/development (Abou-Donia et al., 2019; Tomova et al., 2019; Ayaydın et al., 2020; Zheng et al., 2021). One hypothesis suggests that modified microbiota with elevated levels of pathogenic taxa can evoke immune responses and consequently damage to the intestinal barrier. Another study showed that the serum levels of S100B protein were higher in children with ASD (64 individuals) than in controls (46 individuals) and that these levels were positively correlated with disease severity (Al-Ayadhi and Mostafa, 2012).

Brain-derived neurotrophic factor (BDNF) is a member of the neurotrophic family of factors that also includes nerve growth factor (NGF) and neurotrophic factors 3 and 4 (NT3 and NT4) (Skaper, 2012). BDNF participates in a wide range of neurophysiological processes and is present in almost all regions of the adult mouse brain, with the highest levels found in the hippocampus and cerebral cortex (Hofer et al., 1990). Some of the critical functions of BDNF include regulation of neurogenesis, glycogenesis, and synaptogenesis as well as neuroprotection and control of short- and long-duration synaptic interactions that influence memory mechanisms and cognition (Foltran and Diaz, 2016; Kowiański et al., 2018). These properties may be strictly associated with the dysregulations of BDNF synthesis and impacts on ASD development. Peripheral blood levels of BDNF can be considered a potential biological marker for evaluating individuals with ASD as increasing evidence suggests association between elevated BDNF levels and ASD (Armeanu et al., 2017; Saghazadeh and Rezaei, 2017).

However, this association remains controversial, with some studies reporting reduced serum BDNF levels in individuals with ASD (Taurines et al., 2014; Hashimoto et al., 2006; Kasarpalkar et al., 2014; Francis et al., 2018), whereas a larger number of studies have reported elevated serum BDNF levels in children with ASD compared to typical development (TD) individuals (Miyazaki et al., 2004; Correia et al., 2010; Zheng et al., 2016; Connolly et al., 2006; Ricci et al., 2013; Bryn et al., 2015; Wang et al., 2015).

One feasible explanation for this is that the elevated levels of circulating BDNF in some of the ASD cohorts may represent compensatory response to frequent oxidative and inflammatory stresses, particularly in the context of GBA in ASD. Pro-inflammatory cytokines, such as IL-6 and TNF-α, can modulate BDNF transcription through the NF-κB and CREB signaling pathways. Acute immune activation may transiently upregulate BDNF as a protective mechanism to preserve synaptic integrity under adverse conditions. Additionally, studies on evaluating the BDNF levels in neonates who subsequently presented with ASD revealed inconsistent results (Nelson et al., 2001; 2006; Kasarpalkar et al., 2014; Croen et al., 2008; Abdallah et al., 2013; Skogstrand et al., 2019).

Thus, controversies exist regarding the role of BDNF in the pathophysiology of ASD and its value as a possible marker of this disorder (Calabrese et al., 2014; Porter and O’Connor, 2022).

Another set of molecules that has immune-related associations with neuroinflammation in ASD patterns include RANTES (CCL5) and eotaxin (CCL11). These two molecules are chemokines released by activated T-cells and are recognized as pro-inflammatory cytokines that activate eosinophils, fibroblasts, endothelial cells, epithelial cells, neurons, and glial cells. Studies have provided evidence of elevated serum levels of both mediators in children with ASD, suggesting their involvement in neuroinflammatory processes (Han et al., 2022; Wang et al., 2022).

In another study, the authors reported increased plasma levels of chemokine ligand 5 (CCL5) and 11 (CCL11) in individuals diagnosed with ASD (n = 80) compared with their age- and sex-matched TD controls (n = 58). The authors also noted that children with greater behavioral impairment had significantly higher levels of CCL5 (Ashwood et al., 2011). Han et al. (2022) evaluated 18 ASD individuals with 16 TD controls and noted higher CCL5 levels in the ASD group that worsened evaluation outcomes on the social interaction and communication scales (Han et al., 2022).

In a murine model of ASD, Chen et al. (2020) demonstrated that transplantation of cultured gut microbiota modulated inflammatory chemokine profiles, including CCL5 and CCL11, in parallel with improvements in ASD-related behavioral abnormalities. The authors suggested that microbiota-mediated regulation of immune signaling pathways may contribute to these observed neurobehavioral effects (Chen et al., 2020). After treatment with gut microbiota transplantation, the mice showed significant improvements in CCL5 and CCL11 levels along with improved behavioral outcomes.

Han et al. (2017) compared the patterns of cytokine and chemokine production levels in 23 ASD individuals (6–17 years) and 17 TD individuals; they also investigated the associations between the immunological biomarker levels and severity of outcomes based on behavioral and cognitive symptoms. The study showed lower scores for the cognitive and executive domains in ASD children than TD children; moreover, higher levels of CCL5 were found to be significantly associated with inattention and hyperactivity symptoms in the ASD group than the TD individuals (Han et al., 2017).

The granulocyte–macrophage colony-stimulating factor (GMCSF) released by T cells is another immune-related molecule affecting the inflammatory patterns of the gut in ASD individuals; in the CNS, this cytokine is associated with neuronal and glial differentiation. In pro-inflammatory settings, the GMCSF is produced by T, B, and resident cells; it is a critical regulator of the maturation and polarization of intestinal macrophages. Studies have shown that GMCSF expression in the intestinal environment enhances macrophage microbicidal activity while suppressing wound healing transcriptional programs, including collagen and platelet-derived growth factor (PDGF) production; this suggests that some level of GMCSF signaling may reduce the potential of epithelial tissue healing in the gut mucosa, which could be associated with the damage cycle of dysbiosis and nutritional deficiencies in ASD individuals due to their selective eating habits (Castro-Dopico et al., 2020). In another study, macrophage-derived GMCSF from ASD individuals was found to severely inhibit the dendritic outgrowth of neurons compared to TD individuals (Takada et al., 2024). Although some studies reported low levels of GMCSF in the blood spots of individuals with ASD (Abdallah et al., 2013), higher levels have been observed in the brains of individuals with ASD (Li et al., 2009).

Moreover, studies have reported associations of GMCSF levels with ASD outcomes, where the evidence shows higher peripheral blood levels of GMCSF in ASD individuals. In a study on sixty children (35 ASD and 25 healthy controls) with a 1-year follow-up period, the authors reported a threefold increase in mRNA levels of synthesized cells in the children with ASD compared to the healthy controls, suggesting an association between GMCSF expression and ASD (Ahmad et al., 2020). One feasible explanation for this could be dysbiosis leading to inflammatory responses that consequently lead to GMCSF amplification in the immune system gut.

Studies have shown higher levels of GMCSF, IL-1α, TNF-α, and IFN-α in children with ASD and Gi symptons. Takada et al. (2024) hypothesized the need to study macrophage activation as it could be responsible for the release of GMCSF, which could mediate pro-inflammatory cytokines (IL-1α and TNF-α) associated with dendritic outgrowth impairments in the neurons of children.

Calprotectin is another inflammatory biomarker associated with many gut conditions (Wang et al., 2025). This protein has several features of a good biomarker owing to its stability, assay reproducibility, and low cost for guiding diagnostic and therapeutic decisions (Murray et al., 2023). Calprotectin exists in abundant amounts in the cytosol of neutrophils and represents almost 45% of the total cytosolic stable protein complex (S100A8 and S100A9) (Edgeworth et al., 1991).

However, calprotectin expression could be evoked under inflammatory conditions irrespective of the presence of bacterial antigens (LPS) and inflammatory mediators, such as TNF-α and IL-1β in human monocytes (Suryono Kido et al., 2005). These protein complexes are pivotal to several biological functions, such as cytoskeletal rearrangements to allow leucocyte recruitment (Vogl et al., 2004) and transportation of arachidonic acid to inflammation sites (Klempt et al., 1997). Thus, several conditions and particularly dysbiosis, where the pathogenic bacterial populations of the gut are increased, could be associated with a higher synthesis of calprotectin, driving an acute pro-inflammatory milieu in the gut mucosa (Azouz et al., 2021).

Despite the well-established pro-inflammatory role of calprotectin, a recent systematic review and meta-analysis reported no evidence of increased gastrointestinal (GI) inflammation, as measured by fecal calprotectin levels, in children and adolescents with autism. However, the study reported elevated calprotectin levels in a subset of autistic individuals presenting with comorbid GI disorders (Mathew et al., 2024).

Studies have also suggested that modulation of the gut microbiota could have potential benefits in reducing the exacerbated immune-mucosa responses caused by alterations to the microbiota (Liu et al., 2022; Ugwu et al., 2024).

One of the methods to achieve such positive associations between microbiota modulation and immune-mediated responses is through FMT. However, there is no single study that completely explores the molecular action mechanism of FMT in the mucosal tissue, reinforcing the necessity of more studies to understand these interactions and how they can be used as tools to treat several conditions.

There are various reports on different methods of developing FMT procedures; these works also include techniques for delivering the treatment (e.g., endoscopy and colonoscopy) safely to the recipients following rigorous donor screening and testing.

## 3 Immune mechanistic pathways in dysbiosis

The immune–molecular interactions between the gut mucosal tissue and the microorganisms are still under evaluation. The long-term and low-grade pro-inflammatory patterns in the gut through the GBA is one of the possible mechanisms of addressing the considerable modifications in areas of the brain responsible for mood and behavior, notably the hippocampus and amygdala; it is believed that these changes are caused by pro-inflammatory cytokines that either cross the blood–brain barrier directly or influence the CNS indirectly through systemic circulation (Cryan et al., 2019).

In the absence of genetic and autoimmune conditions, the pro-inflammatory state generated in the gut is attributable to the reduction in commensal microbiota and increase in pathogenic taxa; this condition could lead to increased pro-inflammatory responses.

The immune mechanisms associated with the gut and CNS are related to the pivotal roles of mediators such as IL-6 and TNF-α that are produced and released by several immune cells, which then drive the immune responses modulating the immune cells. These cytokines bind to specific receptors on the target cells to initiate intracellular signaling reactions, e.g., JAK–STAT and NF-κB pathways, leading to changes in gene expression responsible for the pro-inflammatory and cellular functions (Büschgens et al., 2025).

The results of *in vitro* studies show that cytokine binding to specific receptors can directly initiate the JAK–STAT pathway cascade, leading to measurable phosphorylation of the STAT proteins and subsequent gene expression changes (Büschgens et al., 2025). This finding is supported by preclinical studies in which JAK–STAT inhibitors were used to significantly reduce inflammatory markers (Lei et al., 2021), thereby showing a direct inflammatory association between activation/deactivation and inflammatory outcomes.

In children with ASD, FMT therapy primarily aims to restore the microbiota balance by modulating the pro-inflammatory low-diversity patterns. Recent systematic reviews and original studies have demonstrated the impacts of FMT in ASD-like animal models children with ASD (Zhang et al., 2023; He et al., 2026). In one of these reviews, the authors evaluated five studies that reported benefits in reducing the GI and behavioral outcomes in ASD (Zhang et al., 2023). They found that restoring microbial diversity and improving gut barrier function could decrease gut inflammation, which could be associated with improving neuroinflammation.

However, the long-term safety and efficacy of FMT-based therapies require further validation in more animal models to understand the mechanisms involved as well as establish safety protocols and support basis for large-scale controlled clinical trials.

## 4 Preclinical studies of FMT

All treatments available for human use begin with preclinical *in vitro* and animal models studies. The use of FMT has been evaluated in various studies on rodent models under different experimental conditions. Preclinical rodent models evidence the positive roles of FMT in modulating several CNS conditions, such as anxiety, depression, and ASD-like behaviors models.

Rodent models demonstrate that gut microbiota modifications are directly associated with the behavioral presentation of anxiety. In recent studies using germ-free animals, the microbiota-anxiety-prone donors exhibited heightened anxiety-like behaviors, suggesting a link between microbial composition and emotional behavioral aspects. However, the transfer of microbiota from healthy donors to rodents with anxiety-like behaviors led to significant reductions in anxiety-associated behavior patterns accompanied by restoration of microbial diversity and enrichment of beneficial taxa, such *Lactobacillus* and *Bifidobacterium* (Marrocco et al., 2025).

These improved outcomes highlight that FMT could restore the diversity of intestinal microorganisms, reinforce the integrity of the gut barrier, and reduce negative behaviors.

Several studies in animal models have indicated that damaged intestinal permeability is associated with both anxiety and ASD as it could trigger systemic and neuroinflammatory responses (Vellingiri et al., 2022; Bin-Khattaf et al., 2024).

FMT has been shown to enhance the expression of tight junction proteins, including occludin and claudin, thereby reducing intestinal permeability to pathogenic microorganisms and lowering the levels of pro-inflammatory cytokines such as IL-6, TNF-α, and IFN-γ that are frequently elevated in individuals with anxiety and ASD (Kumperscak et al., 2020).

FMT was found to exert remarkable influences on brain neurotransmitter synthesis and release, which are key factors in the pathophysiology of anxiety and ASD. Animal studies indicate that gut microbiota dysbiosis can decrease the levels of important neurotransmitters such as serotonin (5-HT), dopamine, and γ-aminobutyric acid (GABA) in ASD-like mouse models to disrupt mood and emotional balance (Li et al., 2021).

Recently, Wang et al. (2023) reported a valproic-acid-induced ASD-like model (500 mg/kg) in pathogen-free C57BL/6J mice; the ASD-like induced mice developed dysbiosis and metabolite changes related to the serotonergic transmission and glutamatergic neuronal pathways, which could be associated with their behavioral changes. The microbiota analysis showed significant decreases in the genera *Bacteroides* and *Odoribacter*, both of which are recognized taxa associated with the regulation of 5-HT and glutamate transmission in mice. Furthermore, the study showed that FMT from healthy donors to ASD-like mice, increased *Turicibacter* and *Alistipes* populations that are associated with improvements in ASD-related behaviors, suggesting the effectiveness of FMT in addressing ASD-like outcomes (Wang et al., 2023).

Another pre-clinical study demonstrated the effectiveness of FMT in restoring corticosterone levels in a single prolonged stress (SPS) model (Cheng et al., 2025); this outcome also supports the therapeutic potential of FMT in the treatment of anxiety and ASD.

Thus, FMT could help balance the gut microbiota and improve immune responses against pathogenic microorganisms to reduce the associated pro-inflammatory patterns, which could positively influence neurotransmitter synthesis and consequently impact the behavioral aspects of ASD and anxiety (Huo et al., 2017). Table 1 highlights some of the preclinical studies that investigated the role of FMT in ASD-like animal models.

Eventually, the evidence from such preclinical studies could support further clinical evaluations. In this regard, several recent investigations have sought to clarify the mechanisms underlying the immune–mucosal framework in acute and chronic CNS conditions.

**TABLE 1:** Preclinical studies investigating the impacts of fecal microbiota transplantation on ASD animal models.

Study aim	Model	Main findings	References
Evaluate the safety and tolerability of the FMT modification method on microbiota, GI symptoms, and other ASD-like symptoms	Germ-free mice VPA-ASD-model	Mice receiving FMT from ASD-positive donors developed ASD-like behaviors; conversely, FMT from typical development donors improved behaviors	Kang et al. (2017)
Investigate the gut microbiota and therapeutic potential of FMT in improving the microbiome in ASD	ASD model mice	FMT from healthy donors improved serotonergic and glutamatergic synaptic signaling associated with repetitive social behaviors	Wang et al. (2023)
Evaluate the gut microbiota for molecular involvement in the etiology of ASD and associated behavioral changes	Germ-free mice	Fecal microbiome from ASD children led to ASD-like behaviors with microbiota and neurotransmitter metabolism modifications	Xiao et al. (2021)
Study the efficacy of FMT in improving ASD-like symptoms in BTBR mice	BTBR mice	FMT ameliorated the impaired fatty acid metabolism, thereby improving social behaviors	Zheng et al. (2024)
Fecal microbiota transplantation improves memory deficits and social withdrawal in Fmr1 KO mice	Germ-free mice, VPA-induced Fmr1 KO mice	FMT improved ASD behaviors and TNF- α and IBA-1 levels in the cortex and hippocampus	Goo et al. (2020)

FMT, fecal microbiota transplantation; ASD, autism spectrum disorder; BTBR, black and tan brachyury; VPA, valproic acid; Fmr1, fragile X-messenger ribonucleoprotein 1.

## 5 Clinical studies of FMT

Several clinical investigations are available on the role of microbiota dysregulation in neurological conditions; of these, many are focused on GI effects, but recent works have addressed the behavioral clinical manifestations of ASD and their mechanisms (Feng et al., 2024; Hazan et al., 2020).

Overall, the maturation of gut microbiota in children with ASD appears to be slow and diverge over time compared to the TD group (Table 2). Evidence suggests that these children tend to have a greater abundance of non-sporogenic anaerobes and microaerophilic bacteria in their gastric and duodenal samples (Finegold et al., 2002), along with many alterations in the fecal microbiota composition, consistent with reduced levels of *Bifidobacterium* among individuals with ASD (Iglesias-Vázquez et al., 2020; Andreo-Martínez et al., 2022). In addition, Iglesias-Vázquez et al. (2020) reported elevated levels of *Bacteroides*, *Parabacteroides*, *Faecalibacterium*, *Clostridium*, and *Phascolarctobacterium* (Iglesias-Vázquez et al., 2020). However, these increases were not consistently observed in other analyses employing different statistical approaches (Andreo-Martínez et al., 2022).

**TABLE 2:** Clinical studies addressing FMT in ASD.

Condition	Population (N and age)	FMT framework (species and CFU)	Clinical outcomes	References
ASD	85 3–6 years old	*S. thermophilus, B. breve* *B. longum, B. infantis* *L. acidophilus, L. plantarum* *L. paracasei, L. delbrueckii* *9.0* × *10* ^*11*^ *and 4.5* × *10* ^*11*^	Reduction of inflammation, oxidative stress, and ASD symptoms	Santocchi et al. (2020)
	131 4–11 years	*L. plantarum PS128* *3*.0 × 10^10^ for BW < 30 kg 6.0 × 10^10^ for BW > 30 kg	Improvement of intestinal ASD outcomes	Mensi et al. (2021)
	35 3–25 years	*L. plantarum PS128* 6.0 × 10^10^	Improvement of ASD symptoms	Kong et al. (2021)
	80 7–15 years	*L. plantarum PS128* 3.0 × 10^10^	Improvement of ASD symptoms and gastrointestinal signals	Liu et al. (2019)
	22 4–10 years	*L. acidophilus* 5 × 10^9^	Improvement of ASD symptoms	Kałużna-Czaplińska and Błaszczyk (2012)
	30 5–9 years	*B. longum, L. rhamnosus* *L. acidophilus* 5.0 × 10^8^	Improvement of behavioral and intestinal ASD outcomes	Shaaban et al. (2018)
	41 4-11 years	*Galactooligosaccharide* 1.8 g for 6 months	Behavioral improvement	Grossi et al. (2016)
	8 2–11 years	*B. infantis* 2.0 × 10^11^ 5.0–10.0 × day	Improvement of behavioral and intestinal symptoms	Sanctuary et al. (2019)
	13 3–12 years	*L. casei, L. plantarum* *L. acidophilus, L. delbrueckii* *B. longum, B. infantis, B. breve* *S. thermophilus* *1.8* × *10* ^*6*^–3.2 × 10	Improvement of behavioral and intestinal symptoms	Arnold et al. (2019)
	61 2–16 years	*L. fermentum, L. plantarum, L. salivarius DSM 22776* 1.0 × 10^10^	Behavioral improvement	Guidetti et al. (2022)
	18 7–17 years	Standardized human gut microbiota 2.5 × 10^12^ cells/day	Reduction of ASD symptoms	(Kang et al., 2017 ; 2019)
	40 3-17 years		Improvement of ASD and behavioral symptoms	Li et al. (2021)

Given the growing evidence implicating gut microbiota in the pathophysiology of ASD, probiotics have gained attention as a promising therapeutic approach largely owing to their favorable safety profiles and broad acceptance (Lewandowska-Pietruszka et al., 2023).

In their study, Kang et al. (2017) evaluated FMT from healthy donor children to children with ASD longitudinally assess the impacts of the GI system and gut microbiome on ASD-related symptoms; here, the FMT protocol was evaluated in 18 children with ASD (7–16 years of age) for 18 weeks, where the first 10 weeks involved treatment via FMT and the last 8 weeks were used to follow up the outcomes. The authors found significantly increased *Prevotella and Desulfovibrio* populations in the FMT material from the donors.

Furthermore, significant reductions of abnormal stools or constipation were noted in the children with ASD even after 8 weeks following treatment completion (Kang et al., 2017). The ASD behaviors of these individuals reportedly improved following FMT, as evidenced by a 22% reduction in the ASD scores from the Childhood Autism Rating Scale (CARS) evaluations over the treatment duration and even after 8 weeks of treatment termination.

In a report by Zhang et al. (2022) on washed FMT treatment in 49 children with ASD demonstrated a significant reduction in sleep disturbance scores, with no significant differences between the constipated and non-constipated groups based on the Bristol Stools Scale. Additionally, the treatment improved constipation and did not cause deterioration in stool morphology. It also showed beneficial short-term effects on ASD-related behaviors, particularly in the constipated (Zhang et al., 2022).

FMT was also investigated and discussed as a treatment for ASD in children (Li et al., 2021). Here, the authors reviewed five studies (two prospective open-label studies, two retrospective observational studies, and a case report) with several methodological limitations, among which the main drawback was that none of the studies involved randomized controlled trials (Zhang et al., 2023). All studies reported significant post-FMT improvements in the neuropsychological outcomes of ASD. The two prospective open-label studies showed improvements in the Autism Behavior Checklist (ABC) and Social Responsiveness Scale (SRS) scores after FMT treatment (Kang et al., 2017; Li et al., 2021).

Both retrospective observational studies suggested that FMT treatment could improve the symptoms of ASD; here, one of the observational studies reported improvements in the CARS and ABC scores of the constipation group compared to baseline values after the second course of treatment, whereas the other observational study reported significant differences in the ABC and CARS scores between the baseline and end of FMT protocol evaluations (Pan et al., 2022; Zhang et al., 2022).

The case report involved an 18-year-old Chinese male with Asperger Syndrome who suffered from irritable bowel syndrome with 4–5 predominant events of diarrhea over 6 years along with frequent abdominal pain; here, the affected individual received FMT from a 28-year-old healthy male through transendoscopic enteral tubing, which reduced the intestinal symptoms primarily diarrhea and abdominal pain. In this study, the authors found that this treatment altered the structure of the intestinal microbiota as well as its metabolite profile and attributed these changes to increases in the abundance and diversity of microorganisms associated with SCFA production, such as *Roseburia*, *Bifidobacterium*, *Ruminococcus*, *Prevotella*, *and Faecalibacterium* (Huang et al., 2022).

**FIGURE 2:**
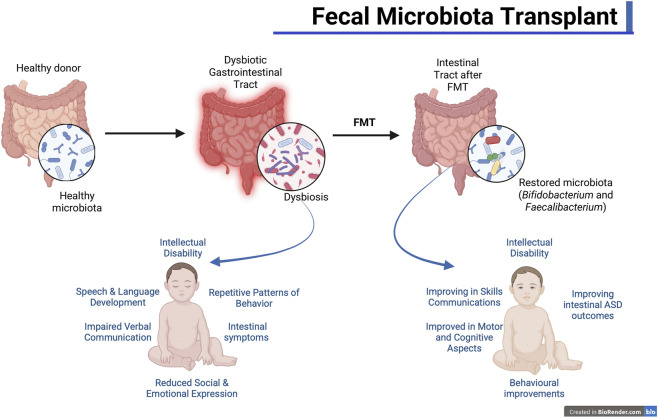
Schematic overview of fecal microbiota transplantation (FMT) as a therapeutic strategy to restore gut microbial balance and modulate the gut–brain axis (GBA). The illustration depicts the transition from a dysbiotic, inflammatory intestinal environment to a more regulated and homeostatic state following FMT. A healthy donor microbiota, characterized by high microbial diversity and an enrichment of beneficial taxa, is transferred into a dysbiotic gastrointestinal tract containing an imbalanced and potentially pathogenic microbial composition. Following FMT, restoration of microbial homeostasis is expected to improve intestinal barrier integrity, reduce local and systemic inflammation, and modulate gut–brain axis signaling pathways. These effects may contribute to improvements in gastrointestinal, immunological, and behavioral symptoms associated with gut–brain axis dysfunction. Created in Biorender.com.

Despite these encouraging reports in the literature, several limitations should be noted regarding the studies. First, the case‐based findings from these studies are very limited, compromising the strength of the data and power of the studies; this reinforces the need for more animal studies and clinical randomized controlled trials. Second, the studies must provide more evidence on the molecular mechanisms and pathways impacting the GBA that drive the microbiota environment modification patterns and *vice versa*. Third, the studies lack patterns or requirements for standardizing the donors recruited for microbiota modifications; this concern is associated with several aspects such as the lifestyle of the donors, such as their diet and exercise habits. Fourth, the donor age could be a potential limitation of FMT for children and young infants owing to differences in anthropometric characteristics, including weight, height, and body mass index. Figure 2 depicts the possible outcomes of FMT in ASD individuals upon restoration of the gut microbial balance.

Collectively, these findings underscore the need for further studies with larger cohorts and more robust analytical frameworks to clarify the gut microbiota alterations associated with ASD.

## 6 Regulatory, ethical, and long-term safety considerations

Despite the recent evidence supporting the therapeutic potential of FMT in neuropsychiatric and neurodevelopmental disorders, several important limitations and challenges remain. Among these, the primary concern is regarding the heterogeneity of FMT protocols, including variations in donor selection criteria, sample preparation, delivery routes, and dosing regimens. These variations hinder reproducibility and comparability of outcomes across studies. Supplementary Figure S2 presents some aspects related to the therapeutic utilization of FMT in neurological disorders, such as ASD.

FMT is inconsistently regulated across different countries and is often classified as a biological product or investigational therapy. In pediatric neurodevelopmental disorders, regulatory oversight is particularly rigorous owing to the vulnerability of the assessed individuals and the limited availability of long-term safety data. Designing clinical trial protocols by adding inclusion and exclusion criteria for individuals with ASD is another major challenge since the tools used for diagnosis and follow-up in children are not consistent across practicing professionals in different countries. Finally, there is limited evidence from randomized controlled trials regarding FMT as a therapy, which raises uncertainty on the benefits and safety for application to the ASD population.

There are significant ethical concerns surrounding FMT in pediatric ASD given the therapeutic misconceptions among parents and caregivers. Since ASD is associated with profound emotional impacts on families, simple participation in such studies could be confused with guaranteed therapeutic benefits. Given the limited availability of effective disease-modifying treatments for ASD and the increasing public visibility of microbiome-based interventions, parents may overestimate the efficacy of FMT or perceive it as a valid therapy rather than an experimental approach. This risk is amplified in neurodevelopmental conditions, where families often seek novel interventions after exhausting conventional behavioral and pharmacological strategies; media coverage, anecdotal reports, and commercial microbiome clinics may further reinforce these inflated expectations.

From an ethical standpoint, this underscores the need for rigorous informed consent procedures that clearly distinguish experimental hypotheses from established clinical benefits. The consent processes should explicitly communicate the current level of evidence, uncertainties regarding long-term outcomes, and possibility of no therapeutic improvement. Independent ethics oversight and structured educational materials may also help mitigate unrealistic expectations and ensure decision-making grounded in balanced risk–benefit appraisal.

Unlike conventional pharmacological agents, FMT involves the transfer of a complex and live microbial ecosystem capable of persistent engraftment within the GI tract of the recipient. This introduces a unique ethical and biological consideration: the potential irreversibility or long-term stability of microbiome alterations following transplantation. Although some degree of microbial resilience is expected, accumulating evidence suggests that donor-derived taxa could establish durable colonization, potentially reshaping the metabolic, immune, and neuroactive signaling pathways over extended periods.

In pediatric populations undergoing critical windows of neurodevelopment, such sustained microbiome modifications raise additional concerns. The long-term consequences of altering microbial–host interactions remain insufficiently characterized, particularly with regard to immune programming, metabolic regulation, and neuroimmune signaling. Furthermore, horizontal gene transfer events, including antimicrobial resistance determinants or virulence-associated traits, cannot be entirely excluded despite rigorous donor screening.

Therefore, FMT in children with ASD must be approached with heightened caution, along with emphasis on long-term follow-up, microbial genomic monitoring, and post-intervention surveillance. Until their durability, reversibility, and downstream systemic effects are understood fully, microbiome-based therapies should be considered as investigational options and implemented within carefully controlled clinical trial frameworks.

## 7 Future perspectives

Future investigations on FMT and related microbiome-based therapies should focus on refining the safety, specificity, and clarity of the underlying molecular mechanisms. One promising direction involves the development of rationally designed microbial consortia or postbiotics, which could provide safer and more controlled alternatives to whole-stool transplantation. Such defined formulations could minimize the risk of pathogen transmission while retaining the therapeutic efficacy through targeted modulation of the host–microbe interactions.

Another emerging avenue is the personalization of FMT strategies guided by comprehensive microbiome profiling, metabolomics, and host genetic data. These precision-based approaches could certainly optimize donor–recipient matching and enhance treatment responsiveness in neuropsychiatric and neurodevelopmental disorders.

The integration of FMT with complementary interventions, including dietary modulation, probiotic or psychobiotic supplementation, and lifestyle-based interventions, could further potentiate and stabilize beneficial microbiome shifts. Finally, well-controlled and well-designed longitudinal studies on humans are essential for determining the persistence of CNS effects, identifying biomarkers, and establishing long-term outcomes. Moreover, the onset of ASD and its immune-associated patterns could be related to other conditions, such as Parkinson’s disease, given that recent studies have shown the link between ASD and the level of inflammatory framework; these findings could also be extended to determine susceptibility to Parkinson’s disease (Zhao et al., 2025).

Collectively, these advancements are critical for translating microbiome science into standardized and evidence-based therapeutic applications for CNS disorders.

## Supplementary material

SUPPLEMENTARY FIGURE S1 Schematic representation of the molecular and immune mechanisms associated with intestinal dysbiosis and epithelial inflammatory damage. Dysbiotic microbiota promotes the release and translocation of pathogen-associated molecular patterns (PAMPs), including lipopolysaccharides (LPS), which activate Toll-like receptor 4 (TLR4) on intestinal epithelial cells. TLR4 stimulation induces downstream inflammatory signaling pathways involving IKK/NF-κB activation and IL-6-mediated JAK/STAT signaling, leading to the transcription and release of pro-inflammatory cytokines, including IL-6, TNF-α, and IFN-γ. The inflammatory microenvironment contributes to increased production of reactive oxygen species (ROS), resulting in mitochondrial oxidative stress and epithelial cell damage. Concurrently, dysbiosis-associated inflammation disrupts epithelial junctional complexes, including tight junction proteins, such as occludin; adherens junction proteins, such as catenin; and desmosomal structures, thereby compromising intestinal barrier integrity and increasing epithelial permeability. Collectively, these mechanisms illustrate how intestinal dysbiosis contributes to mucosal inflammation, epithelial dysfunction, and immune activation.

SUPPLEMENTARY FIGURE S2 This illustration summarizes the major limitations and challenges associated with fecal microbiota transplantation (FMT) as a potential therapeutic strategy for neuropsychiatric and neurodevelopmental disorders. (A) Methodological heterogeneity, including variability in donor selection criteria, stool processing, delivery routes, and dosing regimens, limits reproducibility and cross-study comparability. (B) Placebo responses may be influenced by patient expectations, subjective outcome measures, sociodemographic variables, and behavioral heterogeneity, complicating the interpretation of therapeutic efficacy. (C) The long-term durability, functional stability, and resilience of transplanted microbiota remain insufficiently understood, raising concerns regarding sustained clinical outcomes and the potential need for repeated interventions. (D) Safety concerns include the possible transmission of pathogens, antimicrobial resistance determinants, or undesirable microbial traits due to inadequate donor screening and the lack of fully standardized regulatory frameworks. Collectively, these limitations emphasize the need for harmonized methodologies, rigorous safety monitoring, and long-term clinical studies to establish FMT as a safe, evidence-based therapeutic approach. Created in Biorender.com.
