# Supplementary figures and images for: Immune-related mechanisms of fecal microbiota transplantation in the intestinal microenvironment as a potential intervention for autism spectrum disorder patients
Source: Front Pharmacol. 2026 Jul 8;17:1775104. doi: 10.3389/fphar.2026.1775104 (PMC13388149; doi:10.3389/fphar.2026.1775104)

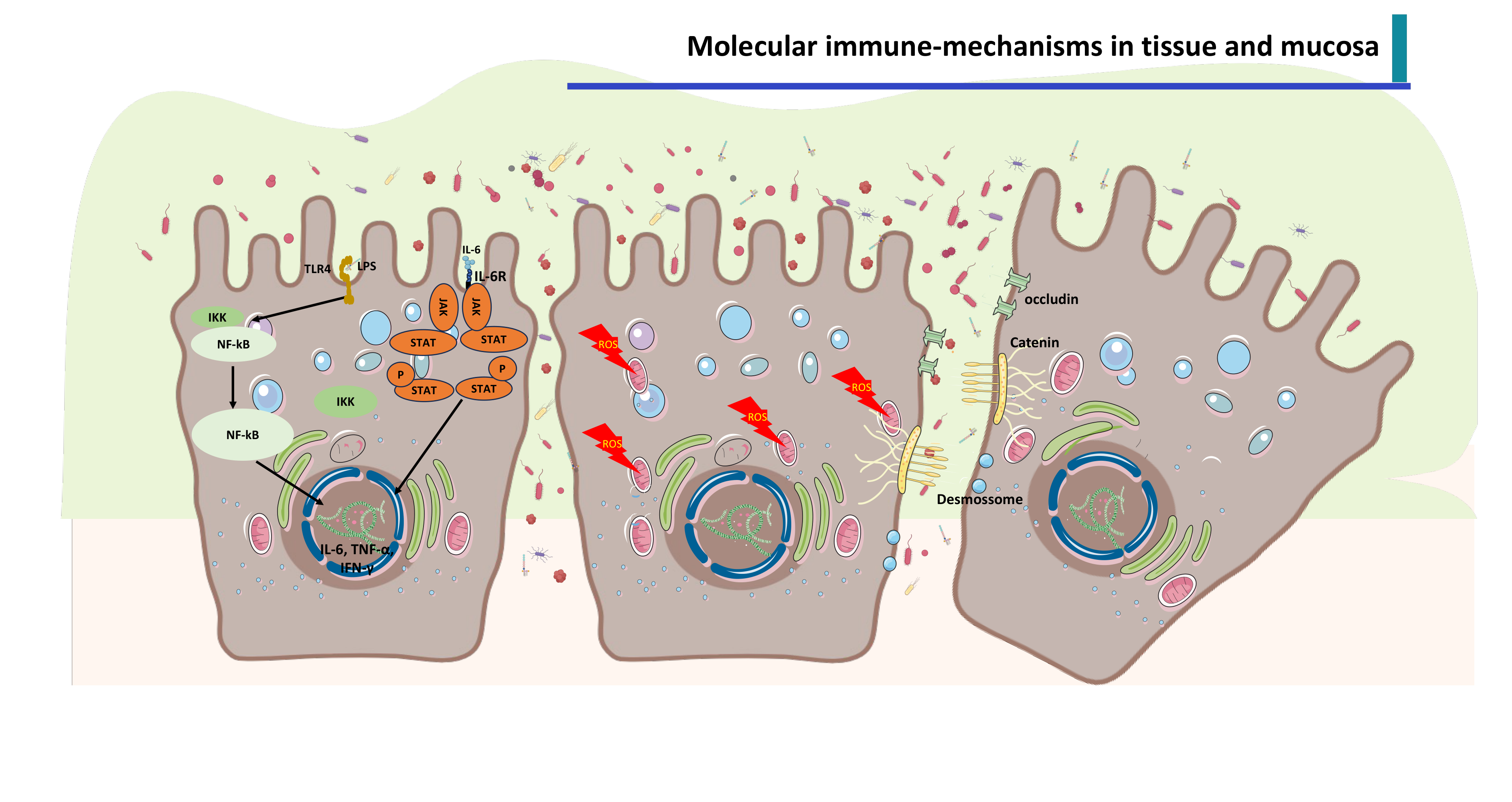

Supplement: Supplementary file 1 [file Image1.tiff]

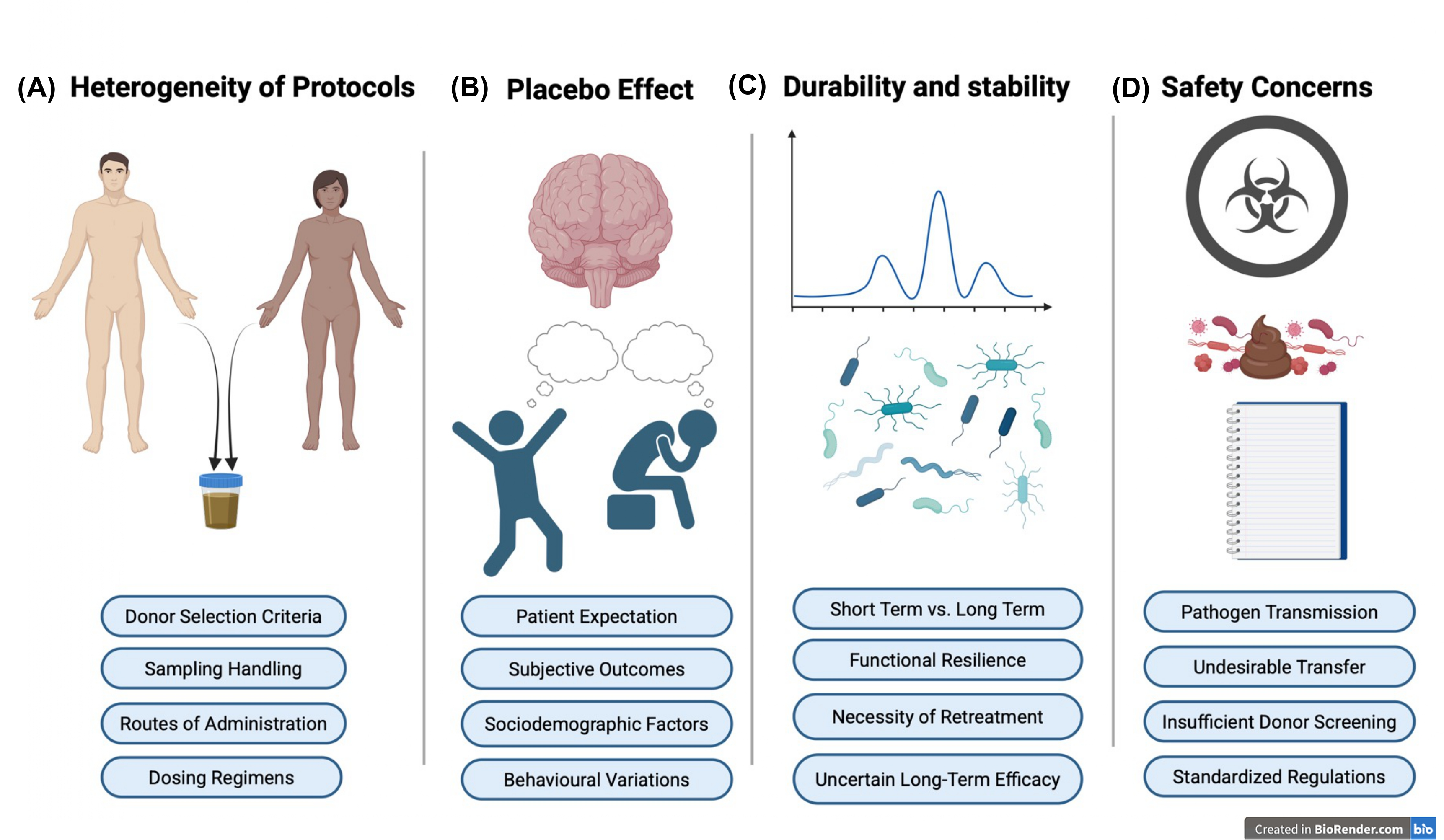

Supplement: Supplementary file 2 [file Image2.tiff]
